# Consciousness: individuated information in action

**DOI:** 10.3389/fpsyg.2015.01035

**Published:** 2015-07-29

**Authors:** Jakub Jonkisz

**Affiliations:** Institute of Sociology, Department of Management, University of Bielsko-BiałaBielsko-Biała, Poland

**Keywords:** taxonomy of consciousness, graded consciousness, extended consciousness, sensorimotor consciousness, integrated information, individuated information, subjectivity

## Abstract

Within theoretical and empirical enquiries, many different meanings associated with consciousness have appeared, leaving the term itself quite vague. This makes formulating an abstract and unifying version of the concept of consciousness – the main aim of this article –into an urgent theoretical imperative. It is argued that consciousness, characterized as *dually accessible* (cognized from the inside and the outside), *hierarchically referential* (semantically ordered), *bodily determined* (embedded in the working structures of an organism or conscious system), and *useful in action* (pragmatically functional), is a *graded* rather than an *all-or-none* phenomenon. A gradational approach, however, despite its explanatory advantages, can lead to some counterintuitive consequences and theoretical problems. In most such conceptions consciousness is *extended globally* (attached to primitive organisms or artificial systems), but also *locally* (connected to certain lower-level neuronal and bodily processes). For example, according to *information integration theory* (as introduced recently by Tononi and Koch, 2014), even such simple artificial systems as *photodiodes* possess miniscule amounts of consciousness. The major challenge for this article, then, is to establish reasonable, empirically justified constraints on how extended the range of a graded consciousness could be. It is argued that conscious systems are limited globally by the ability to individuate information (where *individuated information* is understood as *evolutionarily embedded, socially altered*, and *private*), whereas local limitations should be determined on the basis of a hypothesis about the action-oriented nature of the processes that select states of consciousness. Using these constraints, an abstract concept of consciousness is arrived at, hopefully contributing to a more unified state of play within consciousness studies itself.

“What do I call ‘information that it is raining’? (Or have I only information of this information too?) And what gives this ‘information’ the character of information about something?”

(Wittgenstein, 2009, I. §356)

## Introduction

Over the last few decades, the research into consciousness carried on in both cognitive neuroscience and the philosophy of mind has led to the emergence and consolidation of dozens of divergent meanings for terms central to the topic (Brook and Raymont, 2006; Brook, 2008; Jonkisz, 2012). This polyvalence should surely count as surprising: after all, the resolving of basic conceptual issues would normally be expected to precede the experimental phase of an investigation. With consciousness studies, though, it would appear that questions about the meaning even of the central term itself get to be posed alongside those that pertain to its empirical status. Taking into account this issue alone, the formulation of an abstract concept of consciousness unifying the different branches of consciousness research – the overriding aim of this paper – would seem to represent an urgent theoretical imperative.

An initial step in the direction of meeting that challenge, proposed by the current author, was to assemble a stable framework of connections linking together the numerous varieties of consciousness currently encounterable in science: phenomenal and access, creature and state, sensorimotor and perceptual, reflexive consciousness and self-consciousness, minimal and normal, impaired and altered, clouded and epileptic, visual and auditory, bodily and social, animal and machine, etc. This was accomplished in the form of a proposed *fourfold taxonomy* (Jonkisz, 2012), by showing that virtually all such varieties can be understood as constituted with reference to just four aspects of the phenomenon: namely, its being *dually accessible* (both from the inside and from the outside, subjectively and objectively), *hierarchically referential* (semantically ordered), *bodily determined* (depending on physical states of an organism), and *useful* (possessing diverse pragmatic functions). Ultimately, the varieties of consciousness were accommodated within a taxonomy of *epistemic kinds, semantic orders, physiological state* categories, and the *pragmatically differentiated types* of consciousness supported (see **Table 1**).

**Table 1:** The four-fold taxonomy of consciousness.

Aspects of consciousness		Characteristic	Examples
(Epistemic) Kinds of consciousness	Subjective	Cognized from first-person perspective (experienced)	*phenomenal, first-personal, qualitative, experiential*
	Objective	Cognized from third-person perspective (observed)	*access, psychological, third-personal*

(Semantic) Orders of consciousness	Sensorimotor	Refers to the environment. Enables basic, non-random motor responses utilized, e.g., in space navigation, maintaining equilibrium, etc.	*Sensorimotor, ecological self, proto-self*
	Perceptual	Refers to percepts. Enables identification of the objects of perception and adjusting motor actions	*perceptual, inner sense, first-order representation, primary*
	Meta-perceptual	Refers to perception. Enables multimodal identification of the source of information and adjusting perceptual processes	*HOT, HOP, reflexive, introspective…*
	Self-consciousness	Refers to the perceiving subject. Enables identification of one’s own body and the ownership of the perceptual processes	*self-consciousness, extended, reportable, secondary*
	Meta-self-consciousness	Refers to one’s own self. Enables identification of an owner as an experiencing subject and formulation of the self-concept	*symbolic self-concept, recursive self-consciousness*

(Physiological) States of consciousness	Wakeful	Occur in physiologically normal wakefulness	*NWS, creature, vigilance*
	Sleep-states	Occur in physiologically normal sleep	*sleep, REM-consciousness, NREM…*
	Impaired	Occur in neurological and psychological disorders (e.g., brain lesions)	*LOC, minimal, blurred, clouded, delirious etc.*
	Altered	Occur in ‘non-standard’ conditions (induced accidentally or intentionally)	*ASC, hypnotic, trance, mindfulness meditation, etc.*

(Pragmatic) Types of consciousness	Source-defined	Distinguished according to the type of receptor or sensory system (visual, olfactory,…)	*visual, auditory, tactile, olfactory, gustatory, proprioceptive*
	Use-defined	Distinguished according to the type of situation it is used in	*social, temporal, body, motor-skill, emotional, facial, etc.*
	System-defined	Distinguished according to the type of subject in which it occurs	*animal, human, artificial, machine.*

*Revised version of table introduced in Jonkisz, 2012.

The current article argues that these four taxonomic aspects directly correspond to general or *fundamental features* of the phenomenon. It is also claimed that the broad picture arising from this taxonomy (which includes sensorimotor states within the scope of consciousness) implies a graded rather than an all-or-none nature for the phenomenon. Brief analysis of the empirical status of the graded versus all-or-none (or dichotomous) approaches prompts the conviction that the former is empirically justified, and preferable, when interpreting certain data and medical cases. However, the gradational approach may prove more problematic methodologically (e.g., contrastive analysis is more difficult to obtain; see Baars, 1994; Frith et al., 1999; Overgaard, 2004), and, more importantly, it may generate certain counterintuitive consequences and theoretical problems. Major instances of the latter arise from the fact that in most such theories graded consciousness is significantly extended globally (attached to significantly less developed organisms or even to artificial systems), but also locally (connected to certain lower-level neuronal and bodily processes). For example, according to a recently highly acclaimed theoretical model tailored to the gradational scenario, *information integration theory* (IIT; Tononi, 2004, 2008, 2010; Koch, 2012; Tononi and Koch, 2014), even such primitive artificial systems as photodiodes possess miniscule amounts of consciousness. Hence, one of the major theoretical challenges of this article is to establish reasonable limitations for how extended a graded form of consciousness could be.

The article concurs with the general assumption of IIT that all conscious states are informational states (differentiated and integrated), but above and beyond this the very notion of information will itself be analyzed from a biological perspective (since the phenomenon of consciousness has so far only been known to exist *in vivo*). As a result, information will be characterized as *evolutionarily embedded, socially altered*, and *subjectively grounded* – i.e., *individuated*. The notion of *individuated information* not only fosters a naturalized explanation of subjectivity (potentially rather significant in its own right), but also enables one to impose limitations on conscious systems (the *global limitation postulate*). Subsequently, it will be argued that since not all informational states accessible within a given organism or system are ultimately conscious (see Mudrik et al., 2014), certain additional selection mechanisms should be specified. Because there is no agreement over which of the cognitive functions, neuronal mechanisms, or brain areas are fully responsible for states of consciousness (e.g., attention, memory, language, or certain so called NCC’s, may each of them be neither sufficient nor necessary; see Noë and Thompson, 2004; Lamme, 2006, 2010; Hohwy, 2009), the article seeks to specify which of the general *features of consciousness* is most likely responsible for shaping the selection process. Consequently, one sort of limitation imposed on states of consciousness (the *local limitation postulate*) will be based on an empirically justified hypothesis about the pragmatically functional or action-oriented nature of the selection processes themselves. Finally, taking both local and global limitations as its basis, an abstract concept of consciousness will be arrived at. This conception enables one to link up together certain promising aspects of prominent neurocognitive models (e.g., IIT, GW, GNW) and philosophical ideas (e.g., aspects of ecological, embodied and enactive conceptions), and as such will hopefully contribute to a state of play within consciousness studies that will be (at least conceptually) more unified.

The initial stages of the present enquiry seek to give an adequate characterization of the fundamental features of consciousness. This is followed by analysis of the empirical status of the contrasting all-or-none (dichotomous) and gradational approaches to that phenomenon. Then come sections aimed at setting reasonable global and local limitations on how extended graded consciousness can be. This leads on in turn to the formulation of an abstract concept of consciousness presented in the last part of the article.

## Fundamental Features of Consciousness

We come to know about consciousness in two different ways: by experiencing our own conscious states, and *via* something *else* – namely, observations of certain behaviors and neuronal processes presented by conscious agents. The difference between such subjective and objective forms of knowledge is essentially *epistemic* (hence the category of ‘epistemic kinds of consciousness’ in the abovementioned, previously proposed taxonomy; see Jonkisz, 2012, **Table 1**). Philosophers used to argue that only consciousness and the phenomena it consists of (i.e., thoughts, perceptions, feelings, emotions, desires, sensations, etc.) are subjectively experienced – hence the thought that asking ‘*What-is-it-like* to have it?’ in connection with anything else is absurd. In a similar sense, all or most conscious states are thought to be accompanied by experiential qualities or *qualia* (Nagel, 1974; Jackson, 1982; Dennett, 1988; Searle, 1992, 2000a,b; Block, 1995; Chalmers, 1995, 1996; Kriegel, 2006). As a result, the subjective (*qualitative, experiential, phenomenal*) nature of consciousness stands exposed as absolutely the hardest, and supposedly most enigmatic, aspect of the problem – one that engenders the so called *explanatory gap* faced by scientific approaches, and also perhaps even an *ontologically unbridgeable gulf* (Wittgenstein, 2009; McGinn, 1989; Chalmers, 1995, 1996; Levine, 1983, 2001; Bayne, 2009). Consequently, the distinctive feature of the phenomenon is often taken to be either our split access to it or the fact of its possessing an exclusively subjective character, where such considerations are also then viewed as furnishing a basis for *antireductionist* lines of argumentation and the drawing of *dualistic* consequences. (Such *epistemico-ontological inferences* have been present in the philosophy of mind at least since the time of Descartes’ distinction between *res cogitans* and *res extensa*; see Northoff and Musholt, 2006.) In this article, only the former strategy will be embraced, as the present author holds that *dual epistemic accessibility* does indeed help to distinguish consciousness from various other phenomena observed by science, and so should be regarded as indicative of one of its fundamental features.

Consciousness may be described as a *relation* between a given subject and some particular information or content he or she is aware of or simply possesses. In this sense, it is ‘directed toward’ something or simply ‘refers to’ or ‘is about’ something. It is with much the same considerations in mind that *consciousness* is also labeled either *transitive* (Rosenthal, 1986) or *intentional* (Searle, 1992, 2000a). Yet the latter notions mainly figure in philosophical debates, where ‘intentionality’ in particular, thanks to its ubiquitous deployment across phenomenology, analytical philosophy of perception and philosophy of mind, now seems too complex and ambiguous to be useful (see Pierre, 2003; Siewert, 2006; Lycan, 2014). In this context it is worth adding that consciousness may also refer – and quite often does – to its own content, in that it ‘reflects’ this in higher-order content, creating a hierarchy of orders (in the sense that a given subject X may not only be ‘conscious of Y,’ but also ‘aware of being conscious of Y’). The hierarchy is to a certain extent visible in so called *higher-order theories* of consciousness, or HOT’s (see Rosenthal, 1986; Gennaro, 2005), in various forms of self-directed perception (*introspection* or *inner sense*; see, e.g., Lycan, 1996), and in cases of *metacognition* (see Cohen and Dennett, 2011; Overgaard and Sandberg, 2012; Mealor and Deines, 2013; Shea et al., 2014). More abstract orders of consciousness are known to be correlated with higher levels of information processing in the brain, such as engage progressively more complex neuronal structures and enable the production of more complex forms of behavior: from basic sensorimotor reactions, perceptual differentiations and multimodal coordination to self-identification, verbal reports and the formulation of an abstract notion of selfhood (Hochstein and Ahissar, 2002; Crick and Koch, 2003; Baars, 2012; Palmer and Ramsey, 2012; Baars et al., 2013; Windey et al., 2013, 2014). Simplifying the idea somewhat, consecutive orders of consciousness may refer directly to the environment, to the objects perceived, to the perception itself, to the very perceiver, and/or to the self-conscious subject (see **Table 1** and, for more detailed description, also Jonkisz, 2012). The semantic aspect of *hierarchical referentiality* counts as yet another fundamental feature of the phenomenon.

There exists an extensive body of scientific evidence directly correlating different states of consciousness – alongside their divergent impairments and various other alterations – with specific physiological and behavioral parameters as exhibited by some given conscious agent or other (e.g., Tart, 1969; Teasdale and Jennett, 1974; Bogen, 1995; Metzinger, 2000; Alvarez-Silva et al., 2006; Moller et al., 2006; Kokoszka, 2007; Schnakers, 2008; van Gaal et al., 2011; Långsjö et al., 2012; Panagiotaropoulos et al., 2012; Kouider et al., 2013). In this sense, the claim that all conscious states are determined by the interaction between ‘working’ structures or *subsystems* of a given organism’s body and changing environmental stimuli is quite natural. The fact that consciousness is *bodily determined* counts as another fundamental feature of the phenomenon. The chain of causal effects that are known to influence consciousness is traced back and forth, from the bottom–up molecular and neuronal activations, to the general neurophysiology and, further, to any biological processes or behavioral activities presented by a given conscious body, or specific environmental conditions it is faced with. Limiting ourselves to the biological body proves problematic when taking into account the possibility of non-biological conscious systems like those figuring in the debate over *artificial* or *machine consciousness* (Hollande, 2003; Torrance et al., 2007; Kurzweil, 2012; O’Regan, 2012). It is rather implausible to call states presented by those systems *biological* or *physiological*, yet their consciousness, if they possess it, should share the same fundamental features. In the light of this, it would be less question-begging to say that states of consciousness depend on a variety of physical or structural changes that go on within a conscious system as it interacts with its environment. On the other hand, however, the four state categories featured in the taxonomy (i.e., *wakeful, sleep, impaired, and altered states of consciousness*, see **Table 1**, Jonkisz, 2012) relate directly to the phenomenon of consciousness as it is currently studied in humans or animals, so some reference to the physiology (as a causal intermediary in the larger causal chain) would seem natural.

It seems that consciousness must have been an advantageous trait where the adaptive strategies of our ancestors were concerned (see, for example, Feinberg and Mallatt, 2013). From this perspective, the evolutionary advantageousness of the ability to be conscious may be characterized as *usefulness in action*, in the sense that consciousness fulfills certain *pragmatic functions* – e.g., enabling one to react appropriately, make specific choices, solve new problems, change one’s mind, or plan actions in advance, anticipate developments, etc. It is worth noting here that the notion of *pragmatic function* has much in common with Millikan’s (2004) idea of a *proper function*, as the latter may also be characterized as a form of biologically justified utility common to the creature and its ancestors. One might say that the functions of consciousness are structured and limited by at least three factors: the source of information (type of receptor, sensory, and memory systems), the conditions of application (whether the conscious information is applied to social life, spatial movement planning, or to temporal segregation of events, etc.), and the type of creature or system considered conscious (whether it is human, animal, or machine) – hence the taxonomic division into *source, use*, and *system*-defined *types of consciousness* (see **Table 1**, Jonkisz, 2012). On top of these, *usefulness in action*, defined in terms of *pragmatic functionality*, can then be considered a fourth fundamental feature of the phenomenon of consciousness.

Of course the facts, features, and characteristics ascribed to consciousness are highly varied: for example, that it feels a certain way, is phenomenally structured, is integrated, is a higher-order or self-representational state, is generated by *recurrent processing* in the *thalamocortical structures*, is associated with *reverberating activations* of the *parieto-frontal* areas, that it enables one to behave appropriately, adapt to new situations, learn, understand emotions, differentiate, and choose, etc. (Rosenthal, 1986; Baars, 1994; Block, 1995; Chalmers, 1995; Lycan, 1996; Damasio, 1999; Edelman, 2003; Tononi, 2004; Gennaro, 2005; Kriegel, 2006, 2007; Lamme, 2006; Dehaene and Changeux, 2011, etc.). It is hoped, however, that all of those just mentioned, as well as most other characteristics, ultimately fall into one of the four categories described above, as they concern facts that will prove to be either epistemic, semantic, bodily physiological, or functional. The four fundamental features that enable us to describe consciousness as being *dually accessible*, (*hierarchically*) *referential, bodily determined*, and *useful in action* may therefore be seen as together furnishing an abstract meta-characterization of the phenomenon. Somewhat more precisely, we might say that according to this view, consciousness is an objectively observable and subjectively experienced phenomenon that enables a given agent to utilize increasingly abstract contents. The increasing complexity of the contents and functions of consciousness (depicted in the taxonomic hierarchy of orders – see **Table 1**) is known to be correlated with the higher levels of information processing in the brain that engage progressively more complex, more wide-ranging neuronal structures and significantly more synchronized connection patterns (Hochstein and Ahissar, 2002; Crick and Koch, 2003; Baars, 2012; Baars et al., 2013; Windey et al., 2013, 2014). From this perspective, consciousness shows up as a *graded* rather than *all-or-none* phenomenon (with such gradedness being visible both in the referential contents of consciousness and in the underlying neuronal processes). If certain sensorimotor processes are to be included within its scope (as is assumed in the taxonomy), then the phenomenon also shows up as vastly *extended* among living creatures (since non-random sensorimotor activity is exhibited even by very simple organisms). Just how great the range of organisms is across which consciousness may be said to extend is something that is very much debated in the scientific literature, with few wishing to limit subjective consciousness to humans (Carruthers, 1998) and some ascribing it to quite a variety of non-human animals (Griffin, 2001; Griffin and Speck, 2004; Seth et al., 2005; Edelman and Seth, 2009; Boly et al., 2013; Feinberg and Mallatt, 2013), or even to artificial systems (Hollande, 2003; Torrance et al., 2007; Kurzweil, 2012; O’Regan, 2012). Both the graded character and the range of extension of consciousness are issues analyzed further in subsequent sections here.

## Dichotomous and Graded Consciousness

In experimental practice a given subject is usually only deemed conscious of certain sensory stimuli, perceptual contents, emotions, thoughts, etc., when they (or it) can ‘prove it,’ either through relevant and statistically significant behavioral responses or in the form of conclusive reporting of their own states. In other words, consciousness is equated with either objectively or subjectively measurable forms of awareness. The current debate in science over the adequacy of objective versus subjective *measures of consciousness* (Seth et al., 2008; Overgaard et al., 2010; Sandberg et al., 2010; Szczepanowski et al., 2013; Wierzchoñ et al., 2014) to some extent mimics philosophical arguments over *access* versus *phenomenal consciousness*. Thus, if *phenomenal consciousness* (accompanied by subjective *qualia*) is to indeed be the main and most difficult target of explanation, as some philosophers insist (e.g., Nagel, 1974; Jackson, 1982; McGinn, 1989; Block, 1995; Chalmers, 1995, 1996; Levine, 2001; Kriegel, 2006; Bayne, 2009), then subjective measures would seem to be fundamentally more adequate (Sandberg et al., 2010, 2011; Szczepanowski et al., 2013; Peremen and Lamy, 2014). However, as the methodologies for subjective measuring assume *reportable states* to be decisive, consciousness may seem to be ‘switching on’ from unconsciousness rather late in the process – i.e., not until a given subject is actually able to present such metacognitive reports. Although metacognition, unlike verbal reportability, is not an exclusively human-related ability (Smith, 2009), it is associated with sophisticated stages of information processing, which makes the picture of consciousness appear to be almost exclusively human-related and so rather rare in nature. Often consciousness is viewed not just as coming into play quite late on, but also as doing so suddenly – as an *all-or-none dichotomous* neuronal and phenomenal event (Sergent and Dehaene, 2004; Dehaene et al., 2006). The following sections aim to show that the opposite view, according to which consciousness is a non-dichotomous, *graded* phenomenon, and rather widespread in the animal world, is not only well justified empirically but also explanatorily advantageous (see also Overgaard et al., 2006).

The view of consciousness as dichotomous has garnered substantial support across the field (e.g., Merikle and Reingold, 1998; Merikle et al., 2001; Sergent and Dehaene, 2004; Dehaene et al., 2006; van Gaal and Lamme, 2012; Balconi and Bortolotti, 2013; Landry et al., 2014). There have also been experimental attempts at showing that conscious and unconscious processes are biologically ‘bifurcated’ in that they rely on different neurological mechanisms (Sergent and Dehaene, 2004; Brogaard, 2011; van Gaal et al., 2011; Panagiotaropoulos et al., 2012). It must also be acknowledged that within the currently prevalent methodology of *contrastive analysis* (Baars, 1994; Frith et al., 1999), it is much easier and more effective in practice to demonstrate major differences in neural functioning between higher-order metacognitive states and certain lower-order non-reportable states such as would be considered unconscious, than to detect continuous, small changes, or to show ‘in-between’ states (Overgaard et al., 2006). Even so, the all-or-none picture of consciousness itself is more likely to run into considerable difficulties when it comes to interpreting experimental and medical data. The following cases may be briefly invoked as furnishing pertinent examples: interpretations of Libet-type experiments as examples of strictly unconscious, non-voluntary decision making (Libet, 1985; Soon et al., 2008); cases where the *blindsight* phenomenon is treated as genuinely unconscious perception (Ro et al., 2004; Brogaard, 2011); situations where non-human creatures unable to produce higher-order states are said to be totally bereft of consciousness (Carruthers, 1998); assessments of divergent *impaired states of consciousness* in the dichotomous scenario – according to Giacino (2005), for example, over just 12 years up to 41% of patients inhabiting a *minimally conscious state* were misdiagnosed (see Schnakers, 2008). In all these cases it is debatable whether we are dealing with an absence of consciousness in its entirety or just of certain aspects or higher forms of it (e.g., the ability to report, to discriminate objects, to recall and react accordingly; see below). Positing a dichotomous split between conscious and unconscious processes may also entail certain debatable metaphysical consequences. For example, if a given state does not count as conscious at all until it has become the object of some higher-order or metacognitive state, consciousness may start to look like an extra added property – the sudden by-product of unconscious brain processes, from which *emergentistic* or even *epiphenomenalistic* conclusions might then have to be drawn. So called *conceivability arguments* involving hypothetical *zombie*-creatures (observably identical to us but totally lacking consciousness – see Kirk, 1974, 2005; Chalmers, 1996, 2002; Stoljar, 2007) are clearly much more readily formulated in such a context. With the *all-or-none* picture in mind, consciousness is also more likely to be seen as a certain thing or entity that is either possessed or not (as life was seen by *vitalists*) – something which may eventually prompt one to embrace even more radical *dualistic* consequences regarding its nature. (A more detailed discussion of the metaphysical consequences of the all-or-none approach unfortunately lies well beyond the scope of this article.)

Non-dichotomous approaches, such as portray consciousness as a hierarchical or a graded process, are at least equally popular in the current context of debate (Natsoulas, 1983, 1997a,b; Neisser, 1988; Cleeremans, 1994, 2011; Damasio, 1999; Cleeremans and Jiménez, 2002; Ramsøy and Overgaard, 2004; Tononi, 2004, 2008, 2010; Morin, 2006; Overgaard et al., 2006, 2010; Seth et al., 2008; Kouider et al., 2010; Nieuwenhuis and de Kleijn, 2011; Koch, 2012; Feinberg and Mallatt, 2013; Miller and Schwarz, 2014; Peremen and Lamy, 2014). What is more important, though, is the high level of plausibility those conceptions have, in the light of both considerable supporting evidence and of their explanatory advantages in respect of the interpretation of experimental and medical data – both points to be briefly demonstrated below.

Miller and Schwarz (2014) review the number of behavioral and neurophysiological studies providing extensive support for the gradational view of consciousness, which the authors then apply to the analysis of the decision making process. They conclude that decisions are more likely to be reached in a *gradual process of evidence-accumulation* involving ascending *certainty-levels* (see also Mele, 2010), and a gradually acquired awareness of their being made, than they are to happen suddenly, as products of totally unconscious brain activity with delayed, epiphenomenal consciousness only being acquired later on in the process (Libet, 1985). The results fits well with currently popular methodologies, such as the *perceptual awareness scale* (PAS) proposed in (Ramsøy and Overgaard, 2004), the experimentally supported *continuum of clarity levels* described in (Overgaard et al., 2006, 2010), *post-decision wagering* (Nieuwenhuis and de Kleijn, 2011), and other similar strategies (Massoni et al., 2014; Wierzchoñ et al., 2014).

Further evidence and arguments in support of a hierarchical view of consciousness may be found in neurology, neuroanatomy and psychiatry, as well as in evolutionary and developmental approaches. Gradedness is clearly visible in the way the neuronal processes associated with consciousness unfold. According to the most common view, strong reciprocal connectivity between thalamic nuclei (especially the *intralaminar* and *medial* ones) and the relevant cortical areas enhance gradually more synchronized, more frequent and longer lasting activities in circuits connecting more distant groups of neurons (see, for example, Edelman and Tononi, 2000; Lamme, 2006; Edelman et al., 2011; Baars, 2012; Baars et al., 2013; Saalmann, 2014). The experiments described in Fleming et al. (2010) show significantly higher introspective accuracy in ratings of one’s own conscious decisions in subjects whose brains possess a greater volume of gray matter in the *anterior prefrontal cortex* region. Such sophisticated introspective abilities, realized in the evolutionarily youngest parts of the brain, may be viewed as representing the most advanced grades of self-awareness, and also as revealing their *phylogenetic* immaturity and *phenotypic* divergences in the human brain. Gradedness is also visible in the descriptions of different states of *impairment*. In medical practice the range of impairments and recovery prognoses are assessed against specified scales: e.g., the *Glasgow Coma Scale* and its revisited version (GCS, GCS-R), the *Coma Recovery Scale and Revisited* (CRS, CRS-R), the *Full Outline of UnResponsiveness* scale (FOUR), or the *Wessex Head Injury Matrix* (WHIM; see Giacino, 2005). Patients’ states are analyzed with respect to arousal level and behavioral responsiveness, creating a list of successively *diminished levels of consciousness* with *minimal consciousness* at the end of the list, just before *vegetative state* and *coma* (Teasdale and Jennett, 1974; Giacino, 2005; Schnakers, 2008). Other studies where a non-dichotomous hierarchical approach would seem particularly promising show, for instance, the following: that the return from *anesthesia* also occurs in steps, with so called *primitive consciousness* emerging first (Långsjö et al., 2012); that signs of diminished consciousness are revealed even in severely damaged brains (Philippi et al., 2012); that the *cerebral cortex* is probably not necessary for certain basic forms of consciousness (Merker, 2007); that consciousness occurs, to a certain degree, at different developmental stages (Stuss and Anderson, 2004; Zelazo, 2004); that even very young infants might be perceptually conscious (Kouider et al., 2013); that consciousness is not only a feature of the human mind, as *animal consciousness* most likely extends beyond the *mammalian kingdom* (Lyvers, 1999; Griffin, 2001; Reiss and Marino, 2001; Griffin and Speck, 2004; Seth et al., 2005; Legrand, 2007; Edelman and Seth, 2009; Smith, 2009; Boly et al., 2013; Feinberg and Mallatt, 2013).

*Information integration theory*, as developed recently by Tononi (2004, 2008, 2010), Koch (2012), Tononi and Koch (2014) is an abstract, theoretical model tailored to the gradational scenario: “The IIT claims that consciousness is not an all-or-none property, but is graded: specifically, it increases in proportion to a system’s repertoire of discriminable states” (Tononi, 2008, p. 236). Roughly speaking, according to IIT, a given system is more conscious the more chunks of information it is able to differentiate and bind together or integrate, where “Integrated information (Φ) is defined as the amount of information generated by a complex of elements, above and beyond the information generated by its parts…” (Tononi, 2008, p. 216). Certain problematic consequences and promising aspects of this model will be discussed in the sections below.

## Extended Consciousness and its Limitations

On the basis of the evidence reviewed above, the hypothesis that consciousness is acquired in a graded manner rather than all at once definitely appears valid. If this is the case, it is also likely that at least some of the processes traditionally deemed unconscious rely on the same neurobiological mechanisms as subsequent conscious processes (Peremen and Lamy, 2014), being just an antecedent, lower-level, lower-order, degraded (etc.) form of these. Such an extension of consciousness into processes hitherto considered unconscious receives support, for example, from Overgaard et al. (2008), where the *blindsight* phenomenon is described as a *degraded consciousness* or, even more directly, from Holender and Duscherer (2004, p. 872), where a radical *paradigm shift* is proclaimed by the statement that “…dual-process models, which are based on both conscious and unconscious perception, should be rejected in favor of the single-process conscious perception model.”

Generally, it may be said that in gradational approaches consciousness is most often *extended* below the level of reportable and metacognitive states, to the point where not only lower-order perceptual processes, but also, sometimes, certain non-perceptual processes are reckoned to lie within its scope. Indeed, the latter is the case with this author’s own taxonomic hierarchy of orders (Jonkisz, 2012), in which consciousness is extended beyond perception toward sensorimotor processing. For example, when a given creature effectively avoids obstacles by utilizing visual, tactile, auditory or any other sensory information available in the environment, such functioning might be regarded as an early manifestation of consciousness. In performing such actions with above chance efficiency levels, the creature must be in possession of at least rudimentary sensorimotor information about the object’s surface and location, and about its own body position: on this basis alone it could be said that the creature possesses first-order consciousness of the environment and of its own body, and this even if its conscious states are not available for any *higher-order monitoring* processes (Kriegel, 2007; Jonkisz, 2012). The attribution of consciousness to certain lower-order states may be called *local extension*. By analogy, *global extension* would be an appropriate label to apply when consciousness is being attached to significantly less developed species than humans or primates (Griffin, 2001; Griffin and Speck, 2004; Seth et al., 2005; Edelman and Seth, 2009; Boly et al., 2013; Feinberg and Mallatt, 2013).

In dichotomous approaches, not only are certain non-reportable perceptual states regarded as unconscious (Kihlstrom, 1987; Sergent and Dehaene, 2004; Dienes and Scott, 2005; Dehaene et al., 2006; Scott and Dienes, 2008, 2010; Panagiotaropoulos et al., 2012; Landry et al., 2014), but also certain higher-order processes sometimes get to be placed outside of conscious awareness (van Gaal and Lamme, 2012). Having excluded a great many states, it seems easier for such conceptions to state what consciousness is not than to specify exactly what it is. Meanwhile, in the non-dichotomous gradational views, such as include a whole range of states into the conscious realm, the opposite concern may emerge as equally problematic: namely, the issues of how to determine which states are definitely not conscious. Consequently, no matter which approach is at stake, the methodology of *contrastive analysis* (Baars, 1994; Frith et al., 1999) seems destined to suffer permanently within the science of consciousness, as clear-cut contrasts and precise conditions or strict definitions are still rather difficult to obtain there (Overgaard, 2004). In setting a definite metacognitive or neuronal threshold for consciousness (represented, for example, by reportable states or recurrent processing), dichotomous approaches not only struggle to prove that this is *the* crucial threshold, but also face the burden of providing us with an unambiguous non-operational characterization of what, exactly, it is that is added when the threshold finally gets crossed and consciousness “switches on.” Such a definite borderline for consciousness should be considered dubious on both neuronal and phenomenal grounds: as was already mentioned, it is quite likely that certain perceptual processes traditionally considered unconscious in fact rely on the same neurological mechanisms as those associated with consciousness (Peremen and Lamy, 2014), while there is also strong experimental evidence for gradedness in subjective ratings of conscious contents and decisions with respect to awareness, clarity, certainty, or confidence levels (Overgaard et al., 2006, 2008, 2010; Mele, 2010; Nieuwenhuis and de Kleijn, 2011; Szczepanowski et al., 2013; Miller and Schwarz, 2014; Massoni et al., 2014; Wierzchoñ et al., 2014). In seeking to extend consciousness both locally and globally gradational conceptions struggle, on the other hand, to give unequivocal answers to a number of questions. Which stages of information processing (metacognitive, perceptual, or sensorimotor) form the final frontiers of consciousness? What types of neuronal activity (e.g., *recurrent* or *feed-forward activations*) and which areas of the brain (e.g., only the *corticothalamic complex*, or also certain *limbic* and *midbrain* regions) form the most basic correlates of consciousness? Does consciousness occur only in the mammalian kingdom or across all animal species? What about even more primitive living systems, like plants or bacteria? Could they possess any of its forms? And what about artificial systems (Hollande, 2003; Torrance et al., 2007; Kurzweil, 2012; O’Regan, 2012)? Could any sort of information be accompanied to some degree by consciousness (Chalmers, 1995; Tononi, 2008; Koch, 2012)?

Is it at all feasible to try to impose reasonable, empirically validated constraints on how far graded consciousness might extend? Or could it be that the only option that remains when adopting the gradational approach is a radical extension according to which almost all the information-bearing states of all information-processing systems, either biological or artificial, would be conscious to certain extent? The *double-aspect principle* introduced by Chalmers (1995, 1996), and the IIT proposed by Tononi (2004, 2008, 2010) and Koch (2012), have pursued just this radical strategy. Chalmers (1996) claims that “…wherever there is a causal interaction, there is information, and wherever there is information, there is experience” [by ‘experience’ he means phenomenal consciousness]. He also believes that “Experience is information from the inside; physics information from the outside” (Chalmers, 1996, p. 297 and 305). According to Koch (2012, p. 131)“[a]ny system at all that has both differentiated and integrated states of information…” is more or less conscious. As is commonly known, these theses result in strikingly counterintuitive consequences, such as the attribution of consciousness even to such simple artifacts as thermostats or photodiodes: “Strictly speaking, then, the IIT implies that even a binary photodiode is not completely unconscious, but rather enjoys exactly 1 bit of consciousness” (Tononi, 2008, p. 236). Also rather unpopular are the metaphysical consequences of the claims: i.e., certain forms of *dualism* or *panpsychism* that lead to a reorganization of the ontological structure of the universe with conscious information recognized as another fundamental entity besides *mass* or *energy* (Chalmers and Searle, 1997; Koch and Tononi, 2013; Searle, 1997, 2013a,b).

The authors of IIT have acknowledged the difficulties involved, and have moderated certain aspects of their theory. In particular, they propose a “set of postulates concerning requirements that must be satisfied by physical systems to account for experience” (Tononi and Koch, 2014, p. 4), but Searle’s (2013b) common sense critique of the theory remains sound, at least in some cases. At the same time, Searle might well be wrong where the very notion of information is concerned, this being for him something that cannot be used “to explain consciousness because the information in question is only information relative to a consciousness.” Searle (2013b) seems to be operating with a rather naive or colloquial conception of information (“…information is carried by a conscious experience of some agent (my thought that Obama is president, for example)…;” whereas Koch and Tononi (2013) try to adapt the formal, quantitative conception to the qualitative and unified character of conscious experience. In IIT, information may be approximately described as ‘any difference discernible from a perspective intrinsic to a given system’: “Consciousness… is both integrated (each experience is unified) and informative (each experience is what it is by differing, in its particular way, from trillions of other experiences). IIT introduces a novel, non-Shannonian notion of information – integrated information – which can be measured as ‘differences that make a difference’ to a system from its intrinsic perspective, not relative to an observer” (Koch and Tononi, 2013). If we acknowledge the obvious claim that consciousness consists of states that a given system is able to differentiate from its intrinsic perspective, then we must also agree that consciousness always carries certain information (in the sense of IIT). Consequently, if all conscious states are informational states, then IIT’s notion of information would indeed appear like a good candidate for the general characteristic of consciousness, *contra* Searle. On the other hand, however, the fact that all conscious states bear information does not itself entail that all informational states are conscious – *contra* Tononi (2008, p. 236) and Chalmers (1996, p. 297), for whom information or integrated information is coextensive with consciousness. Such assumptions “strongly violate our intuition…,” as they lead directly to the attribution of consciousness to almost any system possessing informational states – and thus support, in particular, the claim that a thermostat or a photodiode “…feels like something…” (Tononi and Koch, 2014, p. 7).

*Information integration theory’s* notion of integrated information, specifically understood as a simultaneously differentiated and unified state for a given system, fits our intuitions about consciousness, as it seems, indeed, to be composed of such states. However, even if the claim that all conscious states carry some information for the subject is justified and true, it does not necessarily hold the other way around – contrary to IIT, we claim that the question of whether all integrated information is conscious remains to a certain degree open. If we seek to claim otherwise, as IIT itself does (Tononi, 2008), then the characterization of consciousness may be threatened with circularity: ‘consciousness’ is defined in terms of ‘integrated information,’ ‘integrated information’ in terms of ‘differentiated and unified states,’ and ‘differentiated and unified states’ in terms of being conscious. In fact, we may still ask whether it is possible for a given state to be differentiated by a given system, and perhaps integrated, but still not conscious. (That is, we may ask whether it is empirically rather than logically possible; language itself will not, after all, tell us anything new about the real phenomena involved; see Chalmers, 2002). Koch himself (in Mudrik et al., 2014) has recently softened his stance and reconciled himself to the claim that this is indeed the case, at least in four cases: when the *spatiotemporal window* for processing is too narrow, when the level of semantic complexity of the information is low, when sensory information need not be detected multimodally, and/or when certain stimuli or states are already well known and so not novel for the subject. If all conscious states are integrated informational states, but not all states of this sort are conscious, then information integration is not sufficient for consciousness, although most likely it is necessary. Therefore a theory construed in informational terms will have to invoke additional selection mechanisms, or spell out in some other way the special conditions sufficing for consciousness to be present. Hopefully, such a theory will prove feasible and potentially consistent with major empirical findings without obliging one to relinquish the gradational scenario.

The aim of the following sections is to place limitations on what graded consciousness could amount to, thereby avoiding the improbable consequences of IIT’s commitment to radical extension, while nevertheless keeping the notion of ‘information’ in play – as one that remains promising where any general characterization of consciousness is concerned.

### Individuated Information and Global Limitations

If all conscious states are indeed informational states (as was argued above), then it could be worth looking more closely at the nature of information itself. In the context of consciousness studies, it seems reasonable to characterize information from a naturalistic, biological perspective, since up to now the phenomenon of consciousness has only been known to exist *in vivo.*

Biological systems evolve in interaction with their environment and with other creatures; as a result, their bodies and bodily structures are attuned or sensitive to the specific informational resources available in the *ecological niche* of their ancestors (e.g., certain wavelengths of light, acoustic frequencies, or chemicals (etc.) detected on the ground, in the air, underwater, and so on). In this sense, it may be said that the information accessible to a given creature is *evolutionarily embedded* or *phylogenetically determined* – and, as far as we know, it is also *epigenetically modified* across different *phenotypes* (Migicovsky and Kovalchuk, 2011). The ways in which available information is interpreted by living creatures may also be *socially altered*, in the sense of being slightly modified across different groups of individuals even within the same species: e.g., variations in mating habits, ways of communicating danger, ways of hunting, interacting in a group, speaking, etc. (Swaddle et al., 2005). Moreover, the information actually utilized by living creatures is always *subjectively grounded* or *private*, in the sense of being determined by divergent subject-related factors. It is encoded in unique and dynamically changing patterns of neuronal activity, such as are modified by the actual physiological state and/or specific behavioral habits formed during *individual histories* (*ontogenesis*) and shaped by current environmental or social engagements, as well as by the particularities of the relevant location in space. The unique and private form of the informational state a creature experiences will reflect all these specific internal and external conditions – both those currently affecting the biological system at a given moment of time and those that did so in the past.

Such a view partakes of certain important ideas endorsed by *ecological psychology*. According to both Berker (1968) and Gibson (1979), *cognition* is always *situated*: i.e., it belongs to a specific ecological niche and depends on ecological information such as determines the set of possible actions (*affordances*). Perception, in this theoretical approach, is “based on [a given organism’s] detecting information” (Gibson, 1979, p. 1). The biologically focused stance presented here also has a lot in common with the conceptions (Millikan, 1989, 2004, 2006) has introduced. Her notions of ‘biological utility’ and ‘local natural information’ also stress the importance of the usability or applicability of the informational content determined by environmental conditions, by evolution and by individual histories. “The kind of knowledge that earthly creatures have is knowledge applicable in the domains they inhabit, not knowledge for arbitrary nomically possible worlds, nor for other domains, regions or eras within the actual world” (Millikan, 2004, p. 9). *Embodied* and *enactivist* theories also point to and stress similar aspects and determinants of the mind and of consciousness: “consciousness is an achievement of the whole animal in its environmental context” (Noë, 2010, p. 9). In this context, see also (Varela et al., 1991; Clark, 1997, 2008; Hurley, 1998, 2001; O’Regan and Noë, 2001; Thompson and Varela, 2001; Noë, 2006; O’Regan et al., 2006; Engel et al., 2013).

Within such accounts we see information being characterized in the way that has been described here by this author as *evolutionarily embedded, socially altered*, and *subjectively grounded* (*private*). Consequently, the ways in which living systems extract, store or interpret information are determined by their evolutionary antecedents, by interactions with other creatures, and by unique individual histories and actual situations. It may be concluded, therefore, that the form of information functionally relevant for a given biological system is always *individuated* – with respect to just that very system. Yet the philosophical implications of the latter statement are surely fairly momentous, given the *epistemological –* or possibly even *ontological* – consequences that may issue from it. In the former case, it may be claimed that *individuated information* is accessible only from-the-inside, from that system’s own point of view, so that it is *epistemologically subjective*. If one pursues the more far-reaching ontological implications as well, it may be insisted that the *information* only exists inside the system itself, making it *ontologically subjective*. (This is in fact the main point of contention between Searle and Dennett; see Dennett, 1991, 1993, 1995; Searle, 1992, 1995.) There is no need to address that metaphysical issue here, as for current purposes what is crucial is just the very notion of *individuated information*. According to the conception set forth, information functionally relevant for a given biological system is formed in the unique and particular interactions occurring between the system (e.g., its sensory organs located on its body and in relevant areas of its brain) and its environment (e.g., photons, sound waves, chemicals), or just between parts of the system itself, taken in isolation (e.g., its memory subsystems, insofar as these are able to induce certain states internally). Every aspect of the whole formation process possesses its own subject-related uniqueness: from the mere ability to move in certain ways, the sensitivity of its sensory organs, the anatomical and physiological characteristics of certain sensory areas, to its distinct location in some quite specifically ordered spatial milieu at a given moment of time and its individual engagements within a given ecological niche. So we may declare *individuated information* to be subject-related – or, simply, subjective (be it epistemologically or ontologically so).

The evolutionary mechanisms inherited in the context of our terrestrial ecology, and the complexity of the possible interactions between individual entities within it, have resulted in an immense diversity of unique biological systems. As far as we know, every organism is indeed more or less different. For example, even *monozygotic twins* reared together are actually far from identical: developmental changes are known to occur between them at virtually every possible level, be it genetic, epigenetic, anatomical, behavioral, etc. (Pfefferbaum et al., 2004; Fraga, 2005; Ballestar, 2010; Maiti et al., 2011). The same goes for other natural and artificial clones (Freund et al., 2013), as it is inevitably impossible to duplicate all the environmental interactions and internal bodily conditions involved in forming each individual phenotype (at the very least, the exact locations in space of each of a given pair of twins must permanently differ, causing slight differences in stimuli detection, and consequently also in the adaptive plasticity of the processes occurring in sensory areas). Such necessary individual differences, and the uniqueness of biological systems, enable us to account for things like *intrinsic perspective*, the *first-personal point of view*, and the *self*, by furnishing us with a natural source for these, and in fact this sort of *naturalized conception of subjectivity* does seem well justified empirically. Indeed, the major obstacle to creating artificial systems with subjective points of view may well be the combining of these complex biological factors responsible for influencing instances of such naturally formed *perspectivalness*. Does this necessarily mean that all artificial systems are immediately ruled out as potentially conscious (from-the-inside)? Is the ‘information’ utilized by artifacts just *observer-relative* information, as Searle (2013a,b) claims, being information just for us, whereas for artifacts like computers it is just one more piece of binary code being transformed into another? Such conclusions are certainly rather tempting, but our aim here is not to analyze such issues in any detail. We may confine ourselves to remarking that it does not seem fundamentally impossible for artificial systems to individuate information and form private informational states (O’Regan, 2012), though predicting whether it will ever be feasible to construct such systems (e.g., guided by similar biological principles of replication, adaptation, learning, etc.) seems like an issue more suited to futurology. At the same time, the ‘information’ processed by machines based on *computational* (*von Neumann*) *architectures* – even ones as complicated as modern *supercomputers* and *expert systems* – is rather more repeatable than individuated. The situation is somewhat different when *artificial neural networks* are taken into account (Ferber, 1999), though, as these to a certain extent mimic some biological mechanisms (e.g., parallel distributed processing, continuous learning, and adaptation mechanisms, etc.). Yet even such systems do not seem comfortably situated, or to want to fit in specific ecological niches, and neither have they adapted to some specific dynamically changing informational resources – they did not ever evolve so as to act in order to survive, reproduce, and so on, being designed instead just to simulate certain human or animal actions (e.g., face recognition, language production, decision making, etc.). If we still do not know all the necessary and sufficient ingredients of consciousness as a natural phenomenon, then certainly we know even less about the feasibility of any artificial or machine consciousness (Hollande, 2003; Torrance et al., 2007; Kurzweil, 2012; O’Regan, 2012).

Ultimately, however, it seems quite reasonable to restrict graded consciousness to those systems capable of individuating information and, on the basis of this, building subjective points of view. We shall therefore propose the following **global limitation postulate**: conscious experience can only be formed by unique information detectors, i.e., *individual systems*. It is likely that only dynamically changing biological or biologically based systems, evolved and situated somewhere on Earth (or some Earth-like ecosystem), meet these criteria. *Earthly creatures* (Millikan, 2004, p. 44) are able to form individuated informational states which may become conscious at a certain point in the processing that goes on. Our aim in the next section will therefore be to analyze this process more precisely, in the hope of establishing some relevant local constraints on graded consciousness.

### Selection and Local Limitations

If, as was suggested earlier, not all information detected by a given organism and integrated within its specialized *subsystems* (e.g., *primary sensory areas*) is ultimately conscious, then there must be certain mechanisms according to which conscious states are selected. Many hypotheses and empirical findings concerning selection mechanisms at cognitive and neuronal levels have been put forward in science. One of the more promising correlations links consciousness with novel, non-standard or incongruent information (Dehaene and Naccache, 2001; Mudrik et al., 2014). In this context, conscious systems may be seen as specified *difference detectors* and conscious information may indeed be understood as ‘*differences that make a difference*’ – which is to concur with IIT. Roughly put, when the difference is ‘spotted,’ *neuroplasticity* switches on; new associations of neurons are formed and readied to discriminate similar states more efficiently in the future, increasing adaptation, survival rates, and so on. (Hence it is that *learning* and *memory* are often considered crucial functions for consciousness. See also Cleeremans (2011) and Frith (2011). Of course, not all discernible differences are equally likely to be detected: the system turns its *attention* to emotionally, contextually, or perceptually primed contents or stimuli (thus *attention* is also regarded as crucial – see Lamme, 2006). Yet according to Lamme (2006), the phenomenon of consciousness cannot be fully understood just in terms of one function or another: what we should rather be looking for is the specific neuronal mechanism responsible for generating consciousness.

Theoretical proposals and empirical models of neuronal selection most often explore the idea of multiple parallel processes distributed throughout the brain structures that selectively generate states of consciousness (in diverse configurations and different activity patterns). The proposals in question include the *Global Workspace Theory* (GWT; Baars, 1994; Baars et al., 2013), the *Dynamic Core* theory or *theory of neuronal group selection* (DC; Edelman and Tononi, 2000; Edelman, 2003; Edelman et al., 2011), the *Recurrent Processing model* (RP; Lamme, 2006), the *Global Neuronal Workspace* (GNW; Dehaene et al., 1998; Dehaene and Changeux, 2011), or *Semantic Pointer Competition* (SPC; Thagard and Steward, 2014). Borrowing some of their theoretical vocabulary, the process may roughly be described as follows (based on sensory perception): When the sensory information passes a certain detection threshold, *action potentials* in the relevant *feedforward* neuronal networks are fired, enabling one to discern basic features of the sensing scene (crucial for possible motor responses). Later on in the process, longer-lasting activations of higher and lower sensory areas result in the integration of particular aspects of the information into perceptual objects (i.e., localized *re-entrant* or *recurrent processing*). The neuronal activity may then propagate itself onto more distant *corticothalamic* regions, binding these together into wide-ranging firing clusters (*semantic pointers, re-entrant loops*) that may integrate *sensory, motor*, and executive *fronto-parietal* areas. The group that forms recurrent, synchronized activity inside such a DC ultimately wins the competition or ‘survives’ the process of *neuronal selection*, ‘governing’ the neuronal *workspace* for a given length of time (about 100–200 ms, in fact).

It is worth noting that the abovementioned models tend to imply a fairly late and sudden acquisition of consciousness occurring when some particular higher-order cortical activity occurs either more globally (GW, GNW, SPC) or less so (DC, RP), in the *corticothalamic complex*. However, they also reveal certain features that may incline one toward favoring the graded and extended view advocated here (ones also compatible, to a certain extent, with the ideas of IIT; see Tononi and Koch, 2014). For example, in the RP model proposed in Lamme (2006), recurrent activations that operate at a localized corticothalamic level may count as preconscious, or perhaps as conscious but not reportable. The *primary consciousness* conception introduced in Edelman (2003) and Edelman et al. (2011) goes even further in this direction, as it is supposed to be shared by most of the animal world and to precede all higher forms of consciousness (associated with *re-entrant loops* connecting certain memorized emotional values with current sensory inputs). Moreover, in what SPC proposes, consciousness is thought to be significantly extended globally, being associated there with species of gradually diminished complexity in respect of neuronal structure (Thagard and Steward, 2014, p. 79). However, the authors rather arbitrarily assume that fish are the least developed creature possessing consciousness). In addition, the biologically justified picture of consciousness described in recent versions of the GW model (Baars, 2005, 2012; Baars et al., 2013) is also significantly extended globally.

Are we likely to discover mechanisms responsible for selecting states of consciousness amongst some of the lower-level forms of neuronal activity, possibly in the primary sensory and motor areas? Is there any fundamental difference between widespread re-entrant connections and more localized recurrent, or even *feedforward*, firings – except, of course, for their varied spatiotemporal extent, directedness, frequencies, and/or underlying molecular mechanisms? The quantitative methods proposed by IIT may show which of the activity patterns differentiates and integrates more information (Tononi, 2008, 2010; Barrett and Seth, 2011), but since not all integrated information need be conscious (see above and Mudrik et al., 2014), another ingredient will be needed as a basis for the selection. At the same time, however, outside of the varied neuronal characteristics and functional results, we are rather unlikely to find that extra ingredient in the activation patterns themselves. Just putting the matter very roughly, it may be said that widespread re-entrant activations are most likely reportable (when connections reach *prefrontal areas*), and more localized recurrent processing will facilitate the formation of perceptual objects (*percepts*), whilst *feedforward sweep* ‘just’ enables the extraction of basic information necessary for fast motor responses (e.g., location, motion, shape, depth, and color). Nevertheless, the decision as to which of the mechanisms ultimately starts to ‘*generate*’ consciousness in its most rudimentary forms (as its true neural correlate or NCC; see Metzinger, 2000; Noë and Thompson, 2004; Lamme, 2006; Hohwy, 2009) seems always more or less arbitrary, being determined in due course by certain functional assumptions as to whether the subject is able to react appropriately, to differentiate, to learn, to choose, to decide, to recall, to report, etc.

Is it possible, we may ask, to point to any other, perhaps more general, non-neuronal process that might shape selection, avoiding at the same time any arbitrary conclusions about the exact cognitive function or neuronal mechanisms involved? In other words, what might the general difference be between informational and conscious states? The *fundamental features of consciousness* described earlier in the text, understood as abstract meta-characteristics (categories of more detailed features), are good candidates as starting points for this search. Conscious states, according to those four features, are *dually accessible* (subjectively and objectively), *referential* (about something), *bodily determined* (produced by the subject’s physical body interacting with the environment), and *useful in action* (pragmatically functional for a given subject). The question now is this: which of these features are shared by all informational states, and which constitute the *differentia specifica* of consciousness? According to the *global limitation postulate*, consciousness may be engendered only by systems able to *individuate information*. Therefore, all informational states relevant to consciousness must be *individuated* and *subjective*, though not all of them need ultimately be conscious. (This last point in turn raises the issue of *unconscious subjectivity* – a problem that calls for further analysis elsewhere; see Neisser, 2006). Conscious states, as selected-informational states, must share this *constitutive* ingredient; hence, all “consciousness is essentially subjective” (Searle, 1992, pp. 20–21; also Searle, 2000a, pp. 561–570). Both informational and conscious states are not just internally discernible for a given individual system, but also potentially visible to an external observer (e.g., as certain forms of neuronal activity or behavior): in this sense, both are *dually* (subjectively and objectively) *accessible*, even if not accessed at a given moment of time. Things are similar in the case of *referentiality*, since all the *discernible differences* that go to make up informational and conscious states must possess certain content, must be ‘about something’ if they are to be differentiated from one another. (Referential content may exhibit a sensory, emotional, perceptual, or any other sort of character – not necessarily one constitutive of any particular object). To the extent that all informational states are individuated in virtue of being detected by a specifically situated individual body engaged in unique environmental interactions, *bodily determination* should definitely also be considered a common feature. The final candidate for playing the role of a general differentiating element here would seem to be *pragmatic functionality*. The hypothesis that conscious states are selected from informational states by virtue of their *usefulness in action* certainly seems quite viable, so can pragmatic usability furnish us with a *differentia specifica*?

It is a trivial consequence of the mechanisms of evolution that a given creature will be better equipped to detect states if these resemble those that commonly proved useful for its ancestors. It is also evident from the perspective of *developmental psychology* and *neuroscience* that within biologically primed capacities for detecting this rather than that occurrence, once again, the most likely to be used are those states and action patterns resembling the ones that proved most useful during the relevant individual history. So we may assert that informational states are already *pragmatically preselected* by a given creature’s phylogenetic and ontogenetic history, and in that case it must also be rather trivially true to say that the ongoing selection process which ultimately leads to consciousness is *pragmatically driven* in general: i.e., that it is oriented toward selecting from the actions available to a given creature in response to moment-to-moment conditions. In this sense, the real, *in vivo* selecting of states to become gradually more conscious must be shaped by the actual situational context, emotional values, and internal/external conditions that a given system is in. We may add that the selection mechanisms indeed seem to operate as if their aim were to answer the question ‘What can I actually do, having this information?’ rather than the question ‘What do I know?’ (Here we might well wish to invoke such terms as *affordances, contingencies*, or *expectations*; see Gibson, 1977, 1979; O’Regan et al., 2006; Noë, 2006, 2010). Consequently, in this context, the assumption that the major role of consciousness is to govern or coordinate action at a given moment of time seems really quite viable, and in the light of this we can propose the following **local limitation postulate**: that conscious states are informational states selected to coordinate action at a given moment in time.

## Consciousness, Information, and Action

Putting together the global and local limitation postulates just proposed, it seems that the aim of the present paper could after all be fulfilled, and an abstract concept of consciousness arrived at. This conception will concur with the general idea of IIT about the informational nature of conscious states and the graded nature of consciousness (Tononi and Koch, 2014). However, the extended range of graded forms of consciousness will be confined to *individual systems* (i.e., structurally unique, situated creatures, able to individuate information and adapt their actions to changing conditions), whereas differentiated informational states available within a given system are thought to undergo pragmatically driven, action-oriented, complex processes of selection and become, in due course, gradually more conscious. Consequently, consciousness may be characterized quite straightforwardly here: as a phenomenon in which a given *individual system* utilizes information in action. Put more succinctly, **consciousness is individuated information utilized in action**. Of course, such an abstract conception needs to be interpreted in the light of the distinctions and analyses developed here – ones that right from the very beginning have involved the concept of *individuated information* (characterized as *evolutionarily embedded and/or socially altered* and *subjectively grounded*) and a recognition of the fundamental features of consciousness (i.e., *dual accessibility, referentiality, bodily determination, usefulness in action*).

Translating the proposal into more practical terms, it may be said that conscious states consist of all possible sensory, perceptual, emotional, and other (kinds of) contents, selected from internally and/or externally induced informational states (differentiated and integrated in memory-based, sensory, or any other relevant subsystems of a given organism). The contents are selected to coordinate and adapt divergent actions to the specific conditions of the moment: they are utilized, for example, to grasp objects, to navigate motion, to see, to taste, to react appropriately, to decide, to reason, to think, to talk, to read, to answer questions, and so on. Conscious contents actually arise in the very acting process in which different components of actions (i.e., different interacting neuronal and bodily structures) show up as being, in a gradational sense, more or less conscious (leading in turn to a more global kind of task-related integration). Such interacting structures may refer, for example, to specific visual, auditory, tactile, olfactory, gustatory, or inner-bodily sensations, or to emotional feels, or to the location of certain objects in the surroundings, along with their usability, purpose or name, or to co-relations between things and other creatures, or similar past experiences, remembered events and situations, actual thoughts, ideas, concepts, words, phrases, meanings, beliefs, and so on. The degree to which the subject becomes aware of a given content (order of consciousness) is determined by a variety of heterogeneous factors, including general situational and emotional context, the applicability of a given action, the complexity and novelty of the informational states utilized in it, the spatiotemporal range of some given stimuli and the strength of the neuronal activations induced – to name just a few of the possibilities (Ramsøy and Overgaard, 2004; Overgaard et al., 2006, 2010; Mudrik et al., 2014).

Acting in a complex and dynamically changing environment, biological creatures are potentially able to differentiate between an infinite number of states; there can be no doubt that such overloaded informational conditions and such an ever-changing environment would have exerted evolutionary pressure, favoring more efficient selection mechanisms and greater plasticity in acting. The hypothesis that individuated information (with already preselected and subjective states) and consciousness (with action-specific state selection) are adaptive responses to those ‘needs’ seems plausible and consistent with the general scientific picture. The functional role of consciousness implied by this view may be described as that of providing online integration and refinement (adaptation) of available actions in accordance with the changing conditions of the moment. As far as we know, such pragmatically driven processes are most likely probabilistic and heuristic in nature (i.e., experience-based), and rather fallible or susceptible for error, yet nevertheless remain efficient enough to have persisted (Lotto and Purves, 2002; Rossano, 2003; Lotto, 2004). From this perspective, consciousness may be understood as yet another *biological adaptation* – a pragmatically functional process that enables the forming of unique patterns of action coordinated and refined on the basis of the private informational states available to a given agent.

## Consequences and Conclusion

As was argued earlier, one of the characteristic features of conscious states is their referentiality (i.e., being about something). Some of the states, namely higher-order or metacognitive ones, refer to other, namely lower-order, states or contents. In most of the cases, however, a given subject is metacognitively unaware of the particular information utilized in action; economy and efficiency of acting dictate that most of the informational states will go metacognitively unnoticed, being impossible to report for the subject. For example, walking up a hill we are not aware of most of the complex *equilibrioceptive information* that our body uses in order to maintain balance (we just naturally incline into the slope). Perhaps the most controversial claim supported in this article is that even such forms of information, commonly regarded as genuinely unconscious, count as a rudimentary form of consciousness (namely *sensorimotor consciousness*) insofar as they are utilized for the purpose of acting. To put it simply, we *are* our bodies, so if our body ‘knows,’ then we know; in most cases it is just that we do not know *that* we know. One would probably argue that although such sensorimotor information is accessible to the subject, there is ‘nothing it is like to have it’ – no subjective experience in it, no *qualia*. There is insufficient space to analyze the complicated problem of *qualia* here (for that purpose, see Dennett, 1988; Crane, 2000; Bayne, 2009), but it is in fact *subjectivity* that is crucial in this context. The notion of individuated information developed in the present article thus furnishes at least a provisional answer to this objection. It proposes a naturalized conception of subjectivity itself – one based on the uniqueness of biological systems and their individual differences. Extending the analogy just deployed, it may thus be added that we are each of us not ‘*any*-body’: we are, rather, particular, individual bodies, so that no other body could ever ‘know’ anything in exactly the same way that our body does (in that all information accessible within the biological system is individuated). Ultimately, insofar as consciousness is ‘individuated information utilized in action’ by a given creature, what-it-is-like to have individuated information means what-it-is-like for a subject to act (where acting feels a certain way for the subject or is subjectively experienced as such).

In many philosophical theories (e.g., Rosenthal, 1986; Lycan, 1996; Gennaro, 2005) and dichotomous neurocognitive models (Dehaene et al., 2006; Dehaene and Changeux, 2011), consciousness is associated exclusively with certain higher-order metacognitive or reportable states. Except for certain problems and empirical shortcomings (mentioned earlier), such a position also brings the aim of the science of consciousness down to the level of a quest for a certain specific mechanism of generation or, at least, a contrastive threshold between higher-order conscious and lower-order unconscious states. The graded, action-oriented approach developed in this article offers a pragmatic explanation of why there may not be any contrastive difference between conscious and unconscious processes at the neuronal level (see Peremen and Lamy, 2014). The quest for a kind of ‘magic button’ that ‘switches the light on’ in some way or other may be altogether misleading, as the claim that consciousness does not arise suddenly is empirically well justified (as was shown above). According to the view supported here, consciousness shows up in the way in which living systems interact with the world using internal and external information (in this context, see also O’Regan and Noë, 2001; Noë and Thompson, 2004; Noë, 2006, 2010). It builds up gradually in the individual interacting system so that, at a certain point, the system may become able to report on the contents of its own states, yet this does not mean that it has just acquired consciousness while previously having been totally unconscious.

This gradational, action-oriented view also has certain methodological consequences. For example, it is definitely more difficult in experimental practice to find evidence for consciousness at lower levels of functioning – in this case, more sensitive measuring methods and tools are needed. If consciousness is indeed to be action-oriented, then perhaps certain quite new action-oriented measuring procedures will also need to be developed. At the same time, the gradational view does not call for a methodological revolution: for instance, contrastive analysis might also be useful here (when the contrast or threshold is properly defined and detectable). The change, rather, is above all meta-theoretical, because the thresholds or contrasts used in experiments are to be seen more as operational or practical than as definite. Other implications that could have a bearing on methodology might relate to the more *holistic* and *externalistic* approach needed when seeking to explain graded consciousness. A reasonable piece of advice in this respect would be to locate one’s methodology somewhere in between out-of-body experiences and locked-in syndrome (if, that is, we may be permitted to paraphrase the radically externalistic and internalistic methodologies by invoking these well-known states of impairment; see Jonkisz, 2012). It is probably enough just to go ‘*out of our heads*’ (Noë, 2010), toward the most important bodily determinants of consciousness, albeit without necessarily dismissing the fact of the ‘brain’s causal priority’ (Bickle, 2008, p. 272).

Certain consequences of the abstract concept of consciousness developed in this article might prove more significant viewed from a metaphysical perspective, but as detailed analysis is not feasible in this closing section, a few such implications will just be briefly indicated below. Consciousness, understood as ‘individuated information in action,’ is ubiquitous in the animal world: it is definitely not restricted to humans, or to mammals, as its lowest orders might be exhibited by virtually any living system able to make non-random use of environmental information in its actions. What is more, the phenomenon seems to be fundamentally divided epistemologically (because dually accessible), yet can still be, and hopefully is, *ontologically unified*. In the account developed here, consciousness may be seen as implied by or bound to the unique physical structure of any active system that individuates information.

Instead of offering further conclusions, let us end by listing a small number of questions and problems potentially raised by this conception. With such a broad notion of consciousness in play, does the traditional distinction between consciousness and unconsciousness still hold valid? Is it possible for a given creature to act without being conscious, or is acting always information-based, and hence to a certain degree conscious? Is it possible to invent certain criteria for ‘individuation’ in order, for example, to determine whether artificial systems could possibly ever individuate information? How can information be subjective (i.e., individuated) yet not conscious (i.e., not used in action)? What do we mean exactly by ‘action’? Questions such as these, prompted as they are by the conception of consciousness as ‘individuated information in action’ outlined here, certainly merit a discussion of their own.

## Conflict of Interest Statement

The author declares that the research was conducted in the absence of any commercial or financial relationships that could be construed as a potential conflict of interest.
